# Diffusion law of coal gangue slurry and the application of fluidized filling technology of gangue in caving area

**DOI:** 10.1038/s41598-023-38165-y

**Published:** 2023-08-14

**Authors:** Ji-qiang Zhang, Xiang He, Ke Yang, Zhen Wei, Xin-Yuan Zhao, Jue-jing Fang

**Affiliations:** 1 State Key Laboratory of Mining Response and Disaster Prevention and Control in Deep Coal Mines, Anhui University of Science and Technology, Huainan, 232001 China; 2 Institute of Energy, Hefei Comprehensive National Science Center, Hefei, 230031 China; 3 Key Laboratory of Mining Coal Safety and Construction Efficiency of Anhui Province and Ministry of Education, Anhui University of Science and Technology, Huainan, 232001 China

**Keywords:** Civil engineering, Energy infrastructure

## Abstract

In order to deeply study the basic characteristics, diffusion laws, and flow laws of coal gangue and coal gangue slurry, the basic characteristic parameters of coal gangue and coal gangue slurry were obtained through particle size distribution test, electron microscope scanning test, X-ray diffraction test, X-ray fluorescence spectrum test, and angle of repose test. The conveying performance test of coal gangue slurry was carried out, and based on this, a simulation test of coal gangue slurry caving areas was designed. The diffusion and flow laws of coal gangue slurry under the same inclination angle were summarized, and the field test of fluidization filling in the caving areas was conducted. The results show that: (1) The water-to-gangue ratio was the main controlling factor for the conveying performance of coal gangue slurry. The extensibility, slump, and bleeding rate of the coal gangue slurry increased with the increase of the water-to-gangue ratio. (2) The diffusion profile of coal gangue slurry at different dip angles was arc-shaped, and the diffusion distance of slurry increased with the increase of infiltration time. However, there were differences in the sustained diffusion ability of different dip angles. (3) At the same time interval, the spatial accumulation patterns of scattered gangue in different regions will lead to differences in the diffusion speed of the slurry. (4) Both burying and hanging pipes in the falling area can safely and efficiently fill the gangue slurry. The diffusion distance of the caving areas in the test working face was basically consistent with the diffusion distance of the slurry in the simulation test of the coal gangue slurry caving areas.

## Introduction

In 2021, China’s coal consumption accounted for about 56% of primary energy consumption, and the proportion of coal consumption in China will still play a leading role for a considerable period of time^1^. As the main solid waste in the process of coal mining and processing, coal gangue is increasing year by year with the continuous and intensive mining of coal every year. According to statistics, China will produce 729 million tons of coal gangue in 2020. According to coal demand and production forecasts, it is estimated that by 2025, China’s coal gangue production will be about 800 million tons^2^. In 2020, Coal-Thermal Power-Chemical Industry Base in East Ningxia will produce 24.0673 million tons of general industrial solid waste, such as coal gangue, fly ash, desulfurization gypsum, among which 6.091 million tons of coal gangue will be produced. However, due to low calorific value and difficult utilization of coal gangue, it is generally stacked in the open air and dumped on the surrounding surface of the mining area, which is likely to cause environmental pollution such as soil and groundwater^3–5^. At present, the comprehensive treatment technology of coal gangue ground, which mainly focuses on power generation, paving, and building materials production from coal gangue, cannot meet the requirements of the annual growth of coal gangue treatment volume^6–8^. The harmless, resourceful, and large-scale disposal and utilization of coal gangue based solid waste is imminent.

Filling mining can effectively solve the above problems, and has been widely used due to its advantages of high recovery, maintaining stope safety, and meeting environmental needs. It has gradually become an indispensable part of green mining^9–11^. Among them, the fluidization filling technology for waste rock in caving areas is a new filling technology that aims at treating waste rock, simply cementing the broken waste rock or directly adding water to make slurry and pump it into the underground caving goaf^12^. This technology has been successfully tested in the Hancheng mining area recently, achieving the goal of fully utilizing the resources of the underground empty area, thereby achieving the environmental protection goal of not raising the gangue into the well for resource treatment, and achieving the safe transportation of long-distance gangue slurry underground^13,14^. For the disposal and utilization of coal gangue, many scholars at home and abroad have conducted multi-dimensional research from the basic physical and chemical properties of coal gangue slurry^15,16^, diffusion and transportation characteristics^17,18^ and rheological mechanism^19,20^, as well as from the key technologies of fluidized filling technology for gangue in caving areas^21^, and engineering applications^22^. Reference^23^ has studied the basic characteristics of coal gangue and its slurry, the resistance characteristics of slurry transportation, and the flow law in the goaf; A test system for long-distance loop pipe transportation of coal gangue slurry has been designed, the relationship between slurry flow rate and frictional resistance has been established, and the optimal working flow rate and frictional resistance under this condition have been determined; The flow and diffusion test of adjacent grouting filling in the goaf was carried out, and the flow and diffusion law of slurry in the collapse zone of the goaf was mastered. Based on the research on the rheological properties, fluidity, and flow time of fresh coal gangue slurry, the influence of different particle sizes and water-to-gangue ratio on the rheological properties of coal gangue slurry was studied in Ref.^24^, and the changes in rheological parameters, fluidity, and flow time were discussed. Reference^25^ discusses the fluidization diffusion and rock migration laws of coal gangue slurry under different grouting speeds, void ratios, and particle sizes. It is found that the diffusion radius of coal gangue slurry increases with the increase of grouting speeds, void ratios, and particle sizes. The diffusion law of coal gangue slurry is the key and foundation for the smooth implementation and good filling effect of coal gangue fluidization filling technology in caving areas^26^. The above research mainly focuses on slurry transportation, and multiple studies have been conducted on the key issue of slurry transportation, providing a good reference for the diffusion law of coal gangue slurry and the application of fluidized filling technology in gangue falling areas. However, there is a lack of systematic research on the physical and chemical properties of coal gangue and its slurry, the slurry diffusion law of coal gangue particle size and water cement ratio, and the practice of fluidized filling of gangue in the caving area.

In view of this, in order to deeply study the basic characteristics, diffusion laws, and flow laws of coal gangue and coal gangue slurry, this paper obtains the basic characteristic parameters of coal gangue and coal gangue slurry through particle size distribution test, electron microscope scanning test, X-ray diffraction test, X-ray fluorescence spectrum test, and angle of repose test. Carry out conveying performance tests of coal gangue slurry, and explore the impact of different particle sizes and water-to-gangue ratio on the extensibility, slump, bleeding rate, and initial and final setting time of coal gangue slurry. On this basis, a simulation test of coal gangue slurry falling zone was designed to summarize the effects of inclination angle on the diffusion and flow of coal gangue slurry. At the same time, a field test of fluidized filling in the falling zone was conducted in a mine of Ningxia Coal Industry Co., LTD to verify the flow characteristics of coal gangue slurry in the falling zone, and the filling effect was evaluated.

## Basic characteristics of coal gangue and coal gangue slurry

### Particle size distribution, microscopic morphology and mineral composition of coal gangue

**Figure 1:**
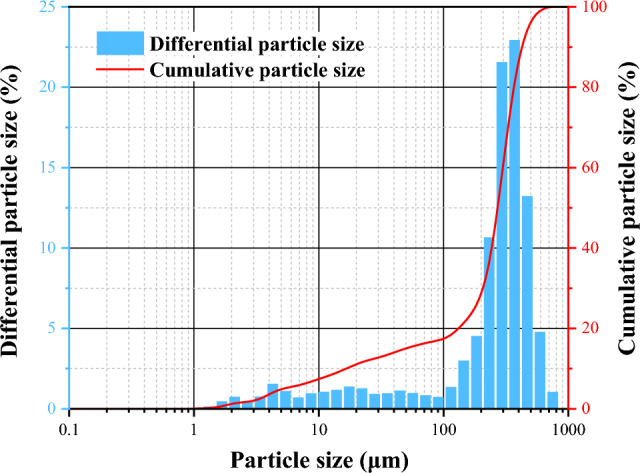
Particle size distribution of coal gangue.

Diffusion test of coal gangue slurry middling coal gangue is taken from a mine of Ningxia Coal Industry Co., LTD. From the appearance, it is dark gray. After screening, it is in granular powder shape, with fineness of 15–25 and loss on ignition of 2–5%; Coal gangue has a certain degree of lubricity, and its particles have no roughness. The particle size test and analysis were carried out by using OMAX dry laser particle size meter, the test results are shown in Fig. 1, the range of coal gangue particle size is generally in 1–1000 μm, the cumulative volume of particle size less than 100 μm accounted for 17.06%, the cumulative volume of particle size less than 500 μm accounted for 94.21%. The volume specific surface area is 0.176 sq.m/c.c., the weight specific surface area is 176.349 m^3^/kg, and the residual error is 0.183%.

**Figure 2:**
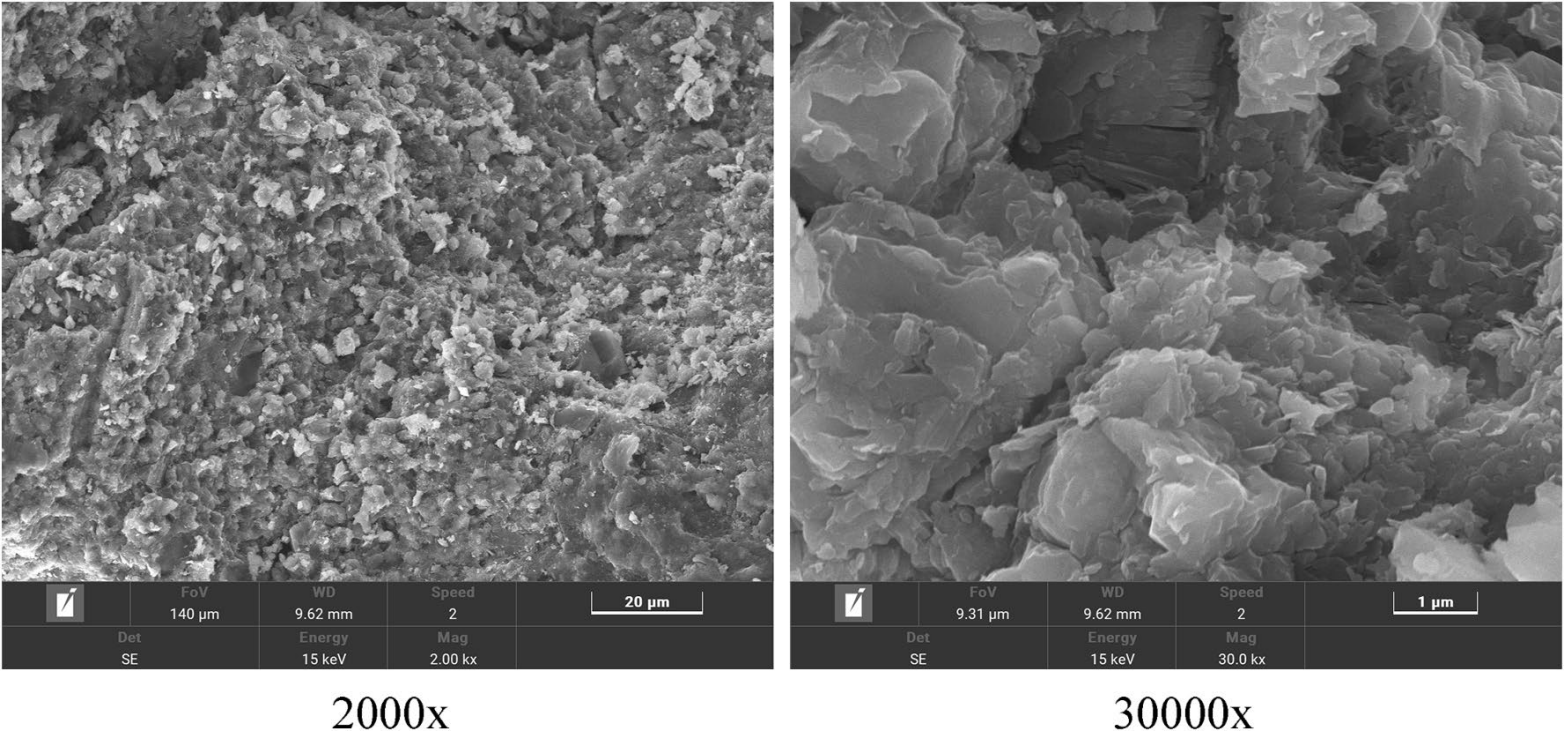
SEM results.

To characterize the micromorphology of crushed gangue powder, an American FEI-Quanta 25 scanning electron microscope was used to test its micromorphology. When observed at high magnification (shown in images at 2000 × and 30000 × ), it was found that there were some small cracks and holes on the surface, which were uneven and presented an irregular polygonal sheet structure. The results are shown in Fig. 2.

**Figure 3:**
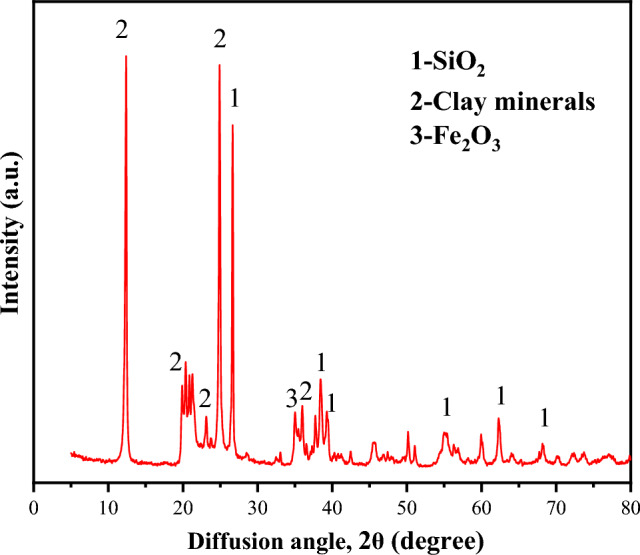
XRD test results of coal gangue.

Quantitative analysis of the whole rock minerals of coal gangue was conducted using a Nippon Nikkei ultima 4 X-ray diffractometer, and the test results are shown in Fig. 3. According to the standard powder diffraction data of various substances provided by the National Data Center of the Powder Diffraction Federation (TCPDS-ICDD), and through comparative analysis using standard analysis methods, the phase composition of coal gangue mainly includes clay minerals, quartz, and a small amount of hematite, as shown in Table 1.

**Table 1:** Statistics of coal gangue phase results.

Sample name	Clay mineral/%	Quartz/%	Hematite/%
Coal gangue	87.8	11.4	0.8

Under the condition of room temperature of 23 °C, the oxide composition of coal gangue was analyzed using the ZSX primus II X-ray fluorescence spectrometer of Nippon Institute of Science. It was found that the main oxide of coal gangue was SiO_2_, with the proportions of Al_2_O_3_ and Fe_2_O_3_ reaching 60.2%, 24.3%, and 5.9%, respectively, as shown in Table 2.

**Table 2:** Statistics of coal gangue XRF analysis results.

Composition	Na_2_O	MgO	Al_2_O_3_	SiO_2_	P_2_O_5_	SO_3_	K_2_O	CaO	TiO_2_
Proportion/%	0.7611	1.2267	24.283	60.1586	0.2602	0.2333	2.8697	2.8818	1.1677

Composition	MnO	Fe_2_O_3_	ZnO	Rb_2_O	SrO	Y_2_O_3_	ZrO_2_	Nb_2_O_5_	Cr_2_O_3_
Proportion/%	0.1256	5.8567	0.0117	0.0125	0.0376	0.0062	0.0512	0.0051	0.0514

According to the scanning electron microscope test results, coal gangue contains a large number of clay minerals (in sheet form), mainly kaolin, hematite, and other minerals, with a large number of “large” sheet shaped particle sizes superimposed and wrapped. During the pulping process, flaky kaolin minerals can be used as fine particles to wrap and suspend large-diameter gangue, which is conducive to ensuring stability and achieving long-term stable transportation.

### Angle of repose test

The coal gangue is crushed by a high-speed multifunctional crusher and divided into particle sizes of 0.2 mm and 0.5 mm through 75 mesh and 35 mesh standard sieves.

The dust continuously falls from the funnel onto the horizontal plate and accumulates into a cone. The angle between the busbar of the cone and the horizontal plane is called the static resting angle of the dust, also known as the angle of repose, (natural) accumulation angle, and placement angle. The angle of repose of dust is related to density, surface area and shape of particles, and the friction coefficient of the substance. It is an important indicator for evaluating dust flow characteristics. Dust with a small angle of repose has good fluidity, whereas dust with a large angle of repose has poor fluidity. The resting angle of 0.2 mm coal gangue is 40.1°, and the resting angle of 0.5 mm coal gangue is 44.1°. This indicates that the larger the particle size, the greater the resting angle, the poorer the flow performance during downhole filling and loop pipe testing, and the more unfavorable for pipeline transportation.

### Coal gangue slurry conveying performance test

#### Test protocol

The main indexes of gangue slurry conveying performance test are slump, extension and water secretion rate. In order to fully study the transportation performance of coal gangue slurry and refer to some relevant literature^27–29^, the gangue slurry water-to-gangue ratio set to 0.5, 0.6, 0.7, 0.8, 0.9, 1.0, 1.1 and 1.2 a total of 8 different water gangue than the test group, each test group were measured its slump, extensibility, water secretion rate, initial and final set time. After the slump measurement, the gangue slurry will be loaded into the standard test mold of 70.7 × 70.7 × 70.7 mm. After filling the mold, natural conditions to dry, to be put into the temperature 20 ± 2 °C, relative humidity of 85 ± 5% of the maintenance box for maintenance, because the maintenance of 1 d and 3 d of the specimen is not formed, so set the maintenance time for 7 d and 14 d. According to Ouattara et al. conclusions^30,31^, the test using micro slump tester (A small truncated cone (one half the size of the standard cone) with height 150 mm, base diameter 100 mm, and top diameter 50 mm was used.) to test the slump of gangue slurry with different water-to-gangue ratio and particle size. The tests were carried out by using the net cement slurry flow test mold (top diameter 36 mm, bottom diameter 60 mm, height 60 mm), measuring tape, volume 250 ml measuring cylinder and vicat apparatus to measure the extensibility, water secretion rate and initial and final setting time of different gangue slurry under different water-to-gangue ratio.

**Figure 4:**
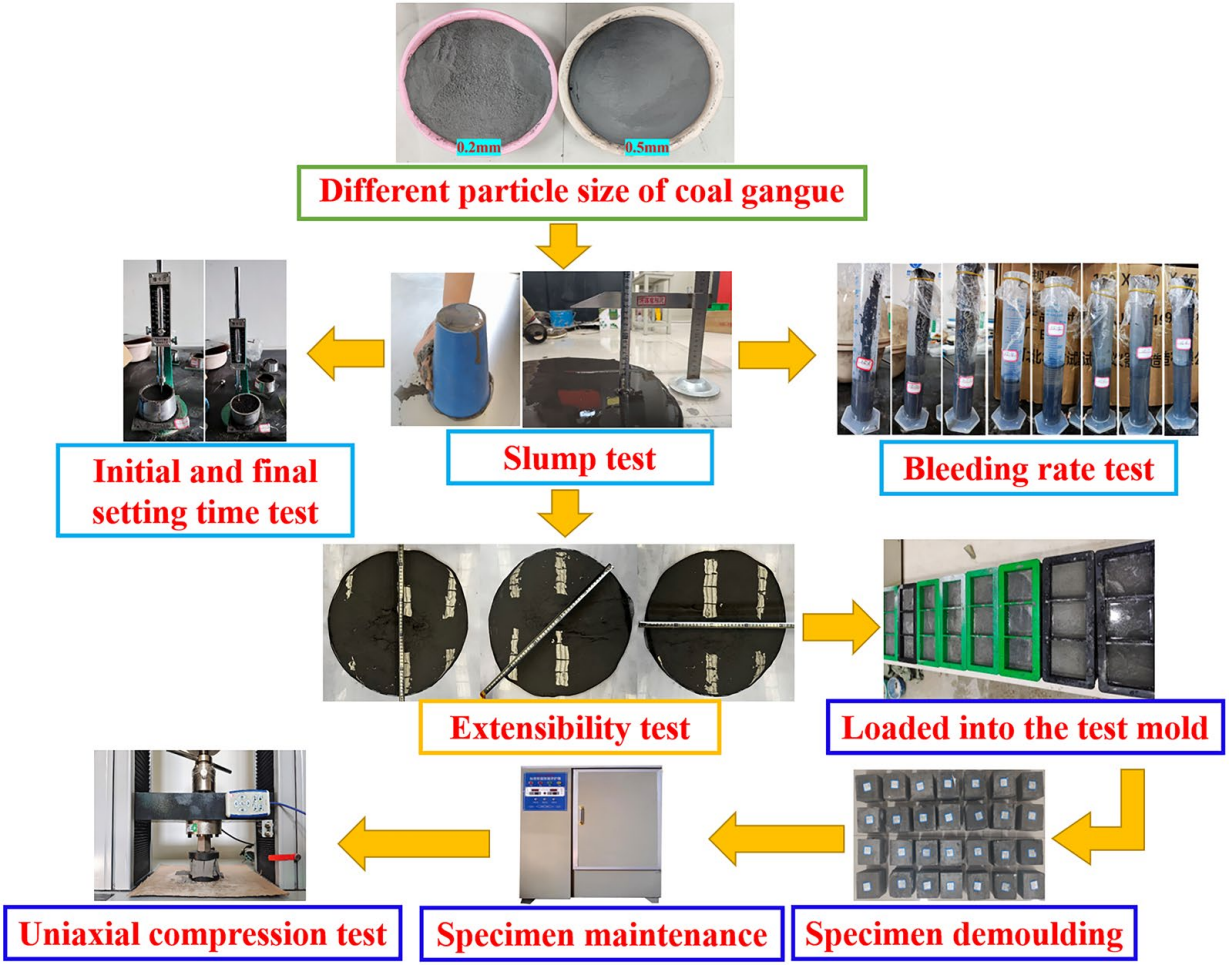
Coal gangue slurry conveying performance test process.

Since there are no standards related to gangue slurry, GB/T50080-2016 national standards were used for the measurement of slump, extension and water secretion rate of different particle sizes (0.2 mm and 0.5 mm) of gangue slurry, respectively. GB/T1346-2011 national standard is used for the measurement of initial and final setting time. According to GB/T23561.12-2010 national standard, the uniaxial compressive strength test was completed on electro-hydraulic servo rock pressure testing machine (loading rate of 0.01kN/s). The specific test scheme is shown in Fig. 4.

#### Test results

Test the slump, extensibility, bleeding rate, and initial and final setting time of coal gangue slurry with different particle sizes under different water-to-gangue ratio conditions. After three tests in each group, take the arithmetic average value of the results, and the results are shown in Table 3.

**Table 3:** Test results.

Water-to-gangue ratio	Slump/cm		Extensibility/cm		Bleeding rate/%		Initial setting time/h		Final setting time/h	
	0.2 mm	0.5 mm	0.2 mm	0.5 mm	0.2 mm	0.5 mm	0.2 mm	0.5 mm	0.2 mm	0.5 mm
0.5	14.40	14.20	13.83	27.67	8.45	16.25	76.43	71.33	93.43	90.20
0.6	14.50	14.25	20.50	29.00	10.89	20.91	77.50	72.45	94.50	91.40
0.7	14.60	14.30	24.33	28.67	18.52	21.93	79.42	74.51	95.42	92.90
0.8	14.65	14.35	27.67	33.33	20.00	25.62	81.46	77.82	97.46	94.60
0.9	14.70	14.40	30.67	34.00	28.04	38.31	83.50	79.40	99.50	96.90
1	14.75	14.50	29.33	27.33	26.67	43.51	85.80	82.50	103.80	99.80
1.1	14.80	14.60	31.00	36.67	33.33	57.50	88.10	88.60	106.10	103.10
1.2	14.88	14.68	32.33	40.33	32.98	59.56	92.48	95.68	109.48	106.30

**Figure 5:**
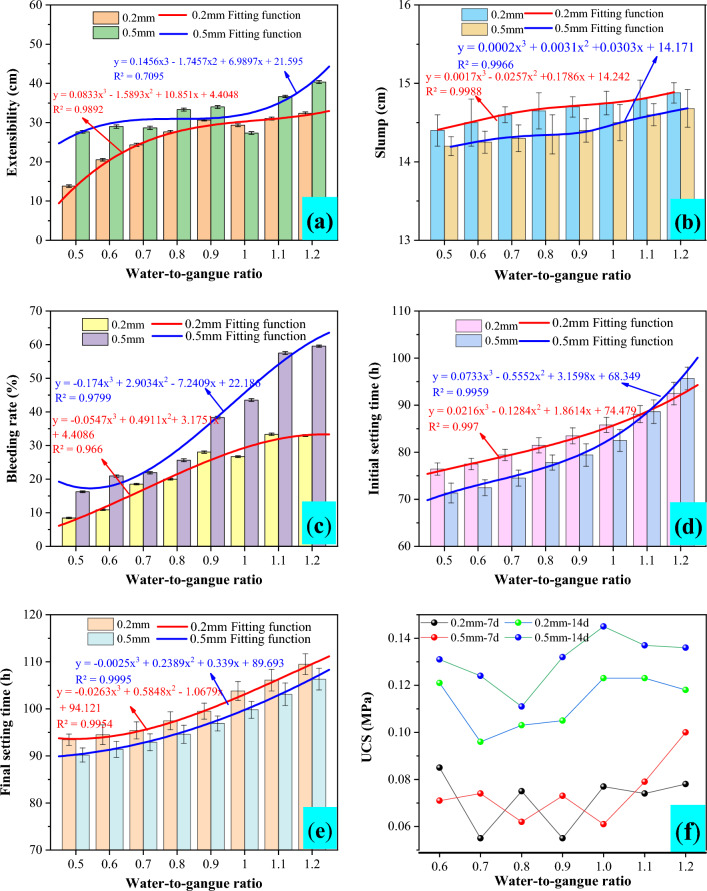
Test results of coal gangue slurry conveying performance.

Polynomial function fitting was performed on the slump, extensibility, bleeding rate, initial setting time, and final setting time of different gangue particle sizes under different water to gangue ratios in Table 3. The results were shown in Table 4 and Fig. 5. From Fig. 5a, it can be seen that under different water-to-gangue ratios, the extensibility of coal gangue slurry with a particle size of 0.2 mm is basically in the range of 13–33 cm, and the extensibility of coal gangue slurry with a particle size of 0.5 mm is basically in the range of 27–40 cm, indicating that the slurry has good flow extensibility. With the increase of the water-to-gangue ratio, the coal gangue slurry with a particle size of 0.2 mm presents a trend of rapid growth at first and then slow growth. The coal gangue slurry with a particle size of 0.5 mm presents a trend of slow growth at first and then rapid growth. When the water-to-gangue ratio is greater than 0.9, the extensibility of coal gangue slurry with a particle size of 0.2 mm increases significantly and slowly, while the extensibility of coal gangue slurry with a particle size of 0.5 mm increases significantly. When the water-to-gangue ratio is greater than 1.2, the extensibility of the slurry increases gradually and steadily. By fitting the expansibility of coal gangue pastes with different water-to-gangue ratio under different particle sizes, the fitting functions for particle sizes of 0.2 mm and 0.5 mm were obtained as *y* = 0.0833*x*^3^–1.5893*x*^2^ + 10.851*x* + 4.4048 and *y* = 0.1456*x*^3^–1.7457*x*^2^ + 6.9897*x* + 21.595, respectively. The correlation coefficients *R*^2^ are 0.9892 and 0.9695, respectively, with a high degree of fit-ting. This function can be used to predict the slurry extensibility under the same conditions.

**Table 4:** Polynomial function fitting results.

Fitted function category	Coal gangue particle size/mm	Fitted function	R^2^
Extensibility	0.2	*y* = 0.0833*x*^3^–1.5893*x*^2^ + 10.851*x* + 4.4048	0.9892
	0.5	*y* = 0.1456*x*^3^–1.7457*x*^2^ + 6.9897*x* + 21.595	0.7095
Slump	0.2	*y* = 0.0017*x*^3^–0.0257*x*^2^ + 0.1786*x* + 14.242	0.9988
	0.5	*y* = 0.0002*x*^3^ + 0.0031*x*^2^ + 0.0303*x* + 14.171	0.9966
Bleeding rate	0.2	*y* = − 0.0547*x*^3^ + 0.4911*x*^2^ + 3.1751*x* + 4.4086	0.9660
	0.5	*y* = − 0.174*x*^3^ + 2.9034*x*^2^–7.2409*x* + 22.186	0.9799
Initial setting time	0.2	*y* = 0.0216*x*^3^–0.1284*x*^2^ + 1.8614*x* + 74.479	0.9970
	0.5	y = − 0.0733*x*^3^–0.5552*x*^2^ + 3.1598*x* + 68.349	0.9959
Final setting time	0.2	y = − 0.0263*x*^3^ + 0.5848*x*^2^–1.0679*x* + 94.121	0.9954
	0.5	y = − 0.0025*x*^3^ + 0.2389*x*^2^ + 0.339*x* + 89.693	0.9995

From Table 3 and Fig. 5b, it can be seen that the slump of coal gangue slurry with different water-to-gangue ratios is basically within the range of 14.2–14.9 m, indicating that there is no obvious accumulation of the slurry. With the increase of water-to-gangue ratio, the slump of coal gangue slurry presents a slow growth trend. When the water-to-gangue ratio is greater than 1, the slump of the slurry increases significantly and slowly. When the water-to-gangue ratio is greater than 1.2, the slump of the slurry basically does not increase significantly. At the same time, it can be found that the impact of the particle size of coal gangue on the slump is relatively obvious. The slump of coal gangue slurry with a particle size of 0.2 mm is significantly greater than that of coal gangue slurry with a particle size of 0.5 mm, and the impact is most obvious when the water-to-gangue ratio is 0.8 and 0.9. By fitting the slump of coal gangue slurry with different water-to-gangue ratios under different particle sizes, the fitting functions for particle sizes of 0.2 mm and 0.5 mm are obtained as *y* = 0.0017*x*^3^–0.0257*x*^2^ + 0.1786*x* + 14.242 and *y* = 0.0002*x*^3^ + 0.0031*x*^2^ + 0.0303*x* + 14.171, respectively, with correlation coefficients *R*^2^ of 0.9988 and 0.9966. The degree of fitting is high, and this function can be used to predict the slump of the slurry under the same conditions.

As can be seen from Fig. 5c, under different water-to-gangue ratios, the bleeding rate of coal gangue slurry with a particle size of 0.2 mm is basically in the range of 8–33%, and the bleeding rate of coal gangue slurry with a particle size of 0.5 mm is basically in the range of 16–60%, indicating a high degree of bleeding of the slurry. With the increase of water-to-gangue ratio, the bleeding rate of different coal gangue pastes presents an increasing trend. When the water-to-gangue ratio is greater than 1.2, the bleeding rate of the slurry still shows an increasing trend. It can be seen that with the increase of the water-to-gangue ratio, the water consumption of the slurry increases, and the bleeding amount also shows a positive increase. The coal gangue has reached a saturated state. By fitting the bleeding rate of coal gangue slurry with different water-to-gangue ratios under different particle sizes, the fitting functions for particle sizes of 0.2 mm and 0.5 mm were obtained as *y* = − 0.0547*x*^3^ + 0.4911*x*^2^ + 3.1751*x* + 4.4086 and *y* = − 0.174*x*^3^ + 2.9034*x*^2^–7.2409*x* + 22.186, respectively. The correlation coefficients *R*^2^ are 0.966 and 0.9799, respectively, with a high degree of fitting. This function can be used to predict the slurry bleeding rate under the same conditions.

According to Fig. 5d and e, under different water-to-gangue ratios, the initial setting time of coal gangue slurry with a particle size of 0.2 mm is basically in the range of 76–93 h, and the final setting time is basically in the range of 93–110 h. The initial setting time of coal gangue slurry with a particle size of 0.5 mm is basically in the range of 71–96 h, and the final setting time is basically 90–107 h. It can be seen that the initial and final setting time of the slurry is relatively long, and the consolidation and hardening speed of the specimen is slow. When the water-to-gangue ratio is less than 1.1, the initial and final setting time of coal gangue slurry with a particle size of 0.2 mm is always greater than the initial and final setting time of coal gangue slurry with a particle size of 0.5 mm. Under the same particle size, the initial and final setting time increases with the increase of the water-to-gangue ratio, which shows that the water-to-gangue ratio has a significant impact on the initial and final setting time of the specimen.

From Fig. 5f, it can be seen that the strength of coal gangue specimens is generally low after 7 and 14 days of curing, basically between 0.05 and 0.15 MPa. Due to the low strength of each group of specimens, the age strength of coal gangue specimens with different water-to-gangue ratios does not exhibit significant regularity. At the same time, with the increase of curing age, the overall strength of coal gangue slurry shows an upward trend, but the increase is not significant.

In summary, the water-to-gangue ratio is the main controlling factor for the conveying performance of coal gangue slurry. The extensibility, slump, and bleeding rate of coal gangue slurry increase with the increase of the water-to-gangue ratio, which indirectly leads to an increase in the initial and final setting time of coal gangue slurry. The extensibility and bleeding rate of coal gangue slurry with a particle size of 0.5 mm are higher than 0.2 mm, while the slump and initial and final setting time of coal gangue slurry with a particle size of 0.2 mm are higher than 0.5 mm.

## Simulation test of coal gangue slurry diffusion in caving area

### Test protocol

**Figure 6:**
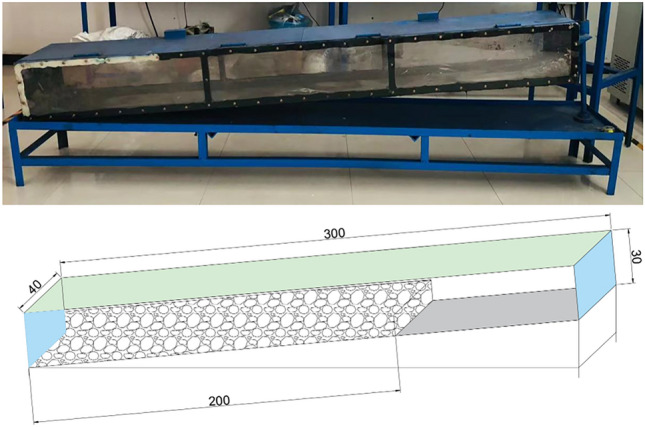
Caving area simulation test box.

Through the conveying performance test of coal gangue slurry, it was found that when the water-to-gangue ratio was 0.9, the extensibility difference between coal gangue slurry with particle sizes of 0.5 mm and 0.2 mm was the smallest, and the slump of coal gangue slurry with particle sizes of 0.2 mm was significantly greater than 0.5 mm, while the bleeding rate of the slurry was less. To sum up, a coal gangue slurry with a water-to-gangue ratio of 0.9 and a particle size of 0.2 mm was selected for the simulation test of the caving area. The test adopts a designed and processed simulation test box with a length, width, and height of 300 cm, 40 cm, and 30 cm in the caving area, which can achieve different angles of coal gangue slurry gravity flow and scattered gangue grouting infiltration tests. The left and right sides of the test box are made of transparent acrylic plates, facilitating the observation and recording of the test process^32^. The simulation test box of coal gangue slurry diffusion in caving area is shown in Fig. 6.

According to the geological conditions of the project site, a simulation test of coal gangue slurry diffusion in caving area with inclination angles of 5°, 7°, 9°, 11° and 13° was designed to simulate and test the flow characteristics of coal gangue slurry under different inclination angles. In the test box, lay loose gangue with a particle size of 30–50 mm to simulate the infiltration and diffusion of coal gangue slurry after filling in the caving area, and observe the infiltration time and distance of the slurry during the test. Before the test, a graded vibrating screen is used to screen gangue with a particle size of 30–50 mm. Each test is strictly weighted according to the material ratio, and the flow distance of the slurry under different inclination angles is taken as the arithmetic average of the three test results.

### Test results

**Figure 7:**
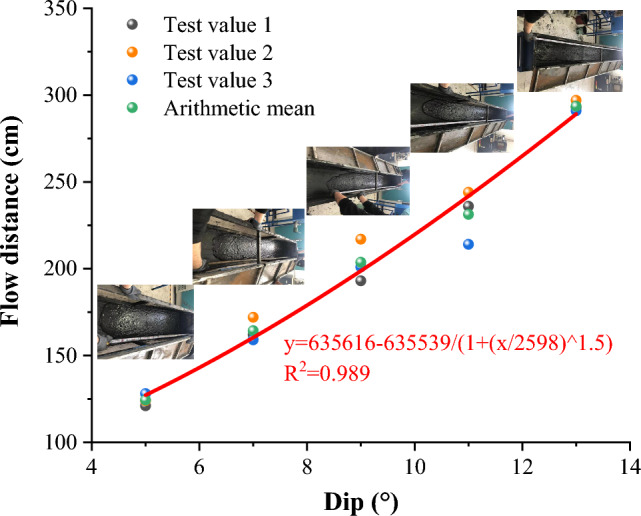
Flow distance of coal gangue slurry at different inclination angle.

The test results of the flow distance of coal gangue slurry under five different inclination angles are shown in Fig. 7.

By simulating and testing the flow distance of coal gangue slurry at different inclination angles, it was found that the flow distance of coal gangue slurry increased with the increase of inclination angle, and the flow range of slurry at different inclination angles reached 41.4–97.9% of the length of the test box. Logistic function was used to perform nonlinear fitting analysis on the flow distance and inclination angle of the slurry. The relationship between the two was a function of *y* = 635,616–635,539/(1 + (*x*/2598) ^ 1.5), with a correlation coefficient of 0.989.

**Figure 8:**
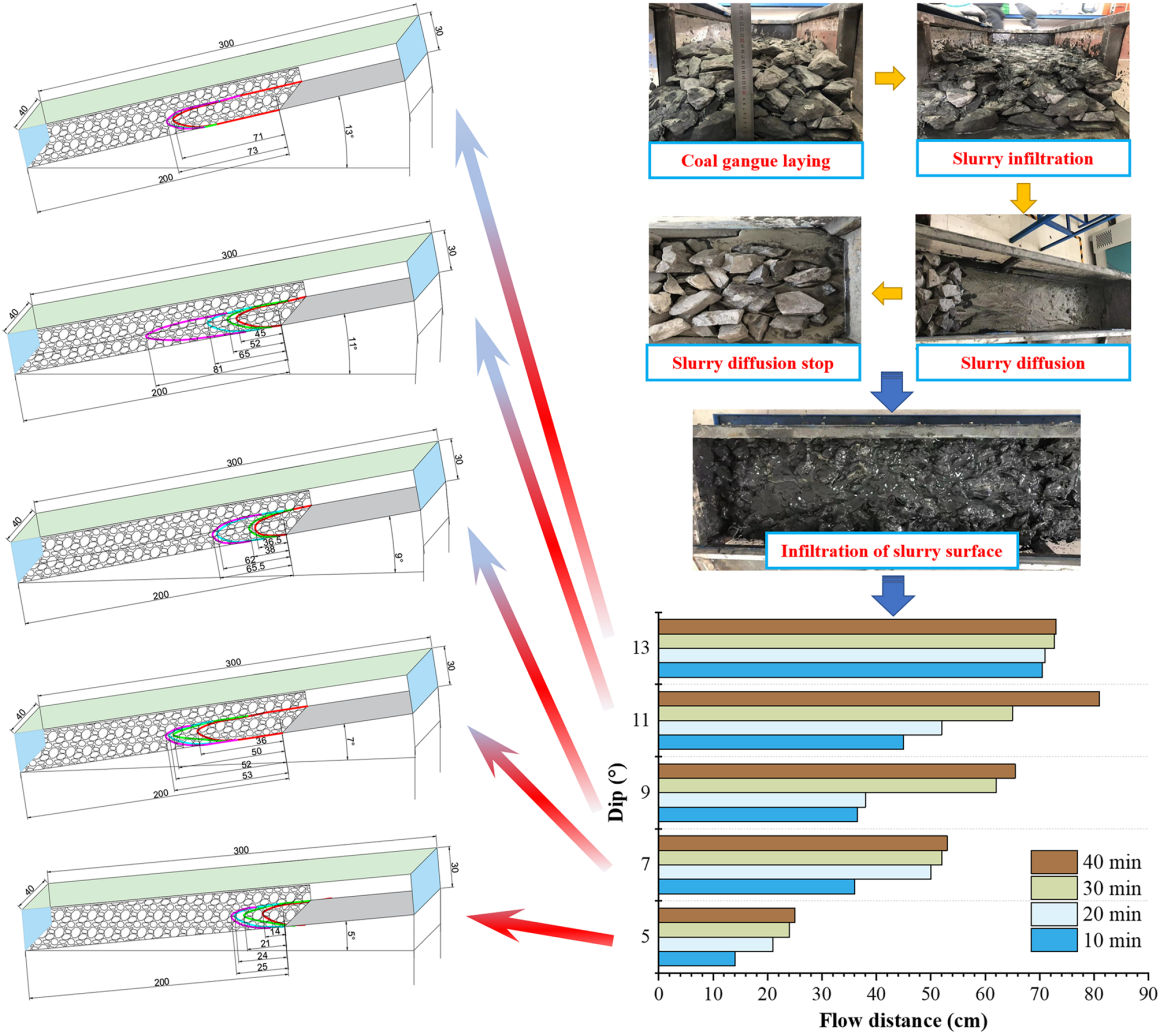
Diffusion evolution process of coal gangue slurry under different inclination angles.

Lay the gangue particles screened before the test into the test box with a thickness of 20 cm and a length of 200 cm. Weigh and prepare a large amount of slurry, adjust the inclination angle of the simulation test box to 5°, 7°, 9°, 11°, and 13°, and slowly and uniformly flow into the bulk gangue pile. Record the diffusion distance and infiltration time of the coal gangue slurry in the gangue pile every 10 min, and draw the slurry diffusion morphology, as shown in Fig. 8.

During the diffusion flow test of coal gangue slurry at different inclination angles, it was found that:

1. The slurry diffusion profile was arc-shaped, and the slurry diffusion distance increases with the increase of infiltration time. However, there were differences in the sustained diffusion ability of different dip angles. The overall diffusion of the slurry at a 5° angle is slower, with a diffusion distance of 14 cm at 10 min, 21 cm at 20 min, 24 cm at 30 min, and 25 cm at 40 min. The overall diffusion of the slurry at a 13° angle was faster, but the continuous diffusion ability was significantly lower than other angles. The diffusion distance was 70.5 cm at 10 min, 71 cm at 20 min, 72.7 cm at 30 min, and 73 cm at 40 min.
2. At the same time interval, there were differences in the diffusion speed of the slurry, which was mainly due to the different spatial accumulation patterns of scattered gangue in different regions, and there were regional differences in the blocking effect on slurry diffusion.
3. The coal gangue had a strong hydrophilicity, and the coal gangue slurry gradually infiltrates and diffuses to the bottom over time, indicating that the slurry had a good fluidity in the interspace of the scattered gangue, which was suitable for grouting and filling the gangue in the underground caving area.

## Field test of fluidized filling in caving area

### Overview of test working face

The 110,904 working face of a certain mine in Ningxia Coal Industry Co., LTD adopts the strike longwall mining method, which completely collapses to handle the roof of the goaf. The average coal thickness is 4.2 m, with an inclination angle of 13°. The working faces are arranged along the coal seam dip, with a strike length of 1192 m and a dip length of 292 m. According to the roof and floor conditions of 119,004 working face, combined with the observation of gangue sampling in the caving area, the false roof and the direct roof of the working face are easy to collapse, and the mudstone, sandy mud-stone and fine sandstone in the gangue in the caving area are severely broken, as shown in Table 5.

**Table 5:** Overview of top and bottom plate of 110,904 working face.

Category	Lithology	Thickness/m	Characteristic
False roof	Limestone	1.3	Gray black, dense and hard
Immediate roof	Mudstone	8.2	Black has a fossilized root and a single delicate structure
Main roof	Siltstone	9.9	The black lithology is single, containing schistose pyrite
Direct bottom	Siltstone	4.4	Gray, grayish purple with thin layers of medium to coarse grained sandstone

### Process flow

**Figure 9:**
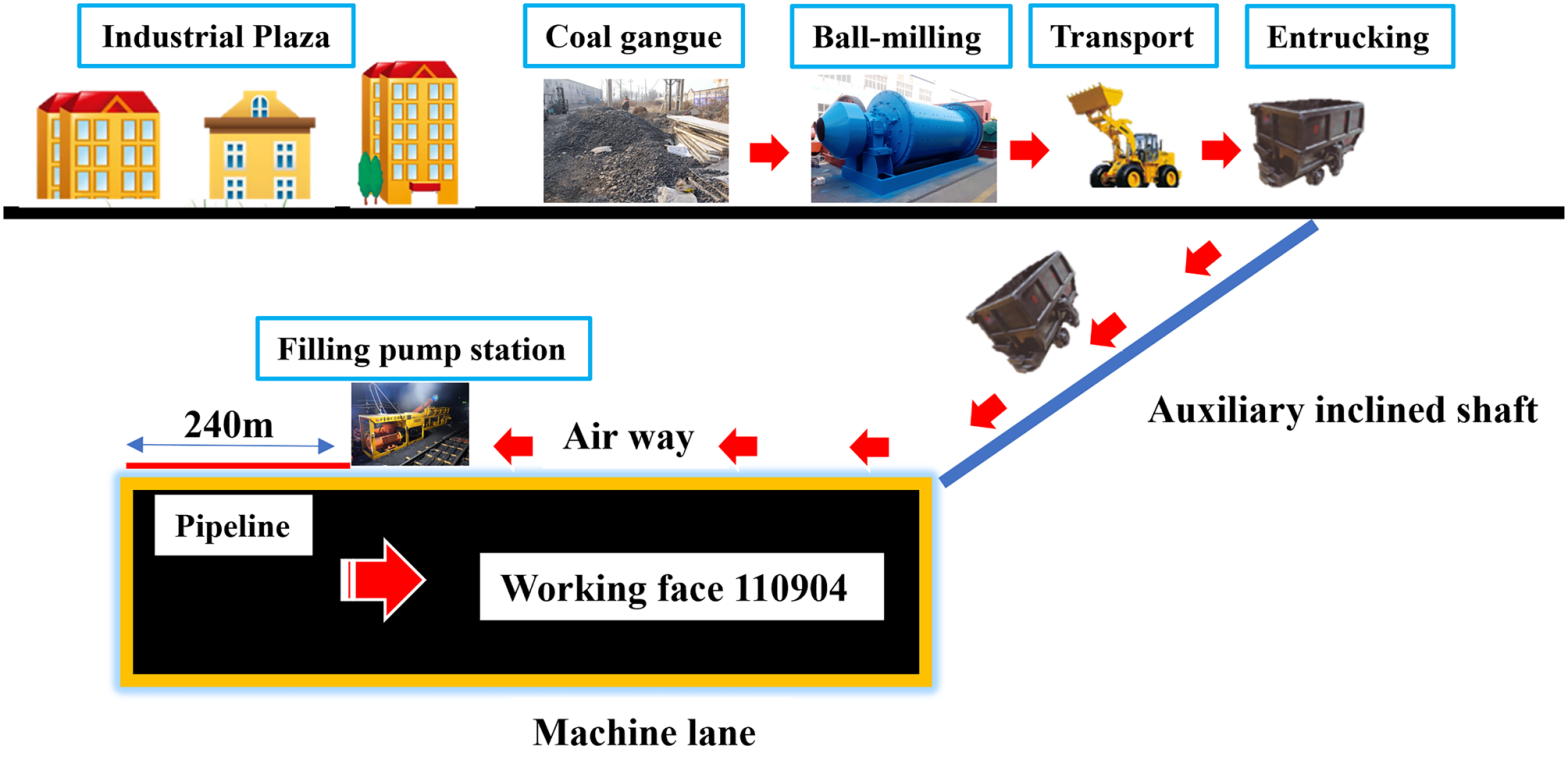
Field filling test process flow.

The fluidization filling system in the caving area mainly includes the waste rock bin on the well, the crushing station, the underground slurry preparation station, the filling pump station, and the grouting pipeline. Used a ball mill in the well to divide the particle size of the coal gangue into 0.2 mm by crushing and screening in advance, and then transport the coal gangue to the underground filling pump station through a tramcar. After weighing the coal gangue in the filling pump station, it was manually fed to a screw feeder, which was transported to a mixer, and water was added to prepare a coal gangue slurry with a water-to-gangue ratio of 0.9. Before grouting and filling, first adjust the grouting pressure to 6 MPa. After stabilizing, pump water for 15 min to moisten the pipe, and then pump cement slurry for 10 min to seal. Then, mix and pump the grouting material. After fully mixing the coal gangue slurry in the mixer, it was finally filled to the caving area through a mining concrete pump. The on-site filling test process flow was shown in Fig. 9.

**Figure 10:**
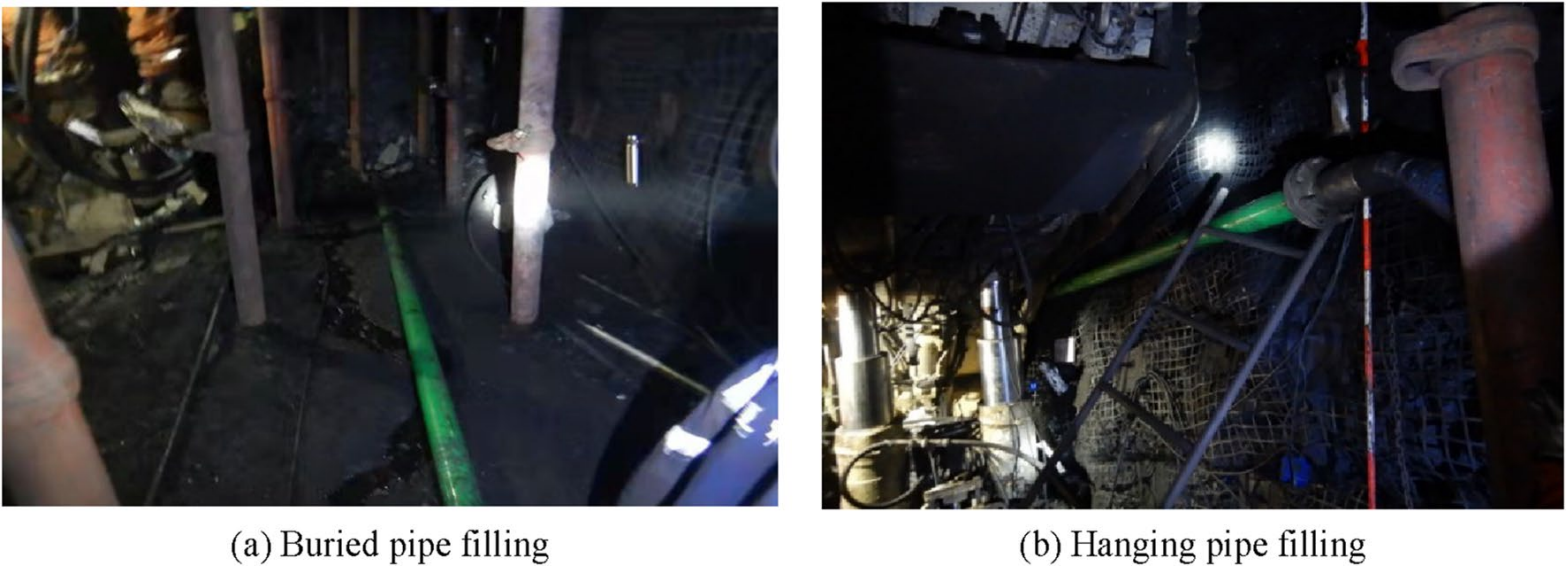
Field test filling method.

The test used HBMG30/21-110S mining concrete pump, with a power of 110 kW, a maximum delivery pressure of 21 MPa, and a maximum delivery capacity of 30m3/h. The mixer model was HBMG3021.11, with a volume of 0.7m^3^. The outlet of the mining concrete pump was Φ125 filling special seamless elbow connection, other used Φ 125 seamless straight pipe for filling. The total length of the Φ125 filling pipeline was 174 m, and the Φ108 seamless steel pipe had a total length of 54 m. A self-made reducer connects the two filling pipes. The outlet of the filling pipeline was located 3 m behind the falling area be-hind the working face frame, ensuring that the slurry was fully filled to the rear of the caving area. In the test, buried pipe filling and hanging pipe filling were used for fluidization filling in the caving area. Buried pipe filling refers to laying the filling pipeline on the bottom plate of the working face, as shown in Fig. 10a. Hanging pipe filling refers to hanging the filling pipeline on the side guard plate of the support, with the pipe outlet extending into the caving area, as shown in Fig. 10b.

### Test results

Three times of roadway pipe filling tests were conducted in the return air roadway of 110,904 working face in a mine of Ningxia Coal Industry Co., LTD, including buried pipe filling once and hanging pipe filling twice, as shown in Table 6.

**Table 6:** Statistics of filling amount of coal gangue slurry.

Filling method	Number of tests	Grouting time/min	Filling slurry/t	Average flow rate/(m/s)	Orifice pressure/MPa
Buried pipe filling	1	50	8	1.4	0.5
Hanging pipe filling	2	20	5	1.5	0.1

Based on the analysis of the test data, the following conclusions were reached:

1. Buried pipe filling and disposal of 8t coal gangue, grouting water consumption of 7.2t, grouting time of about 50 min, average slurry density of 1.54 g/cm^3^, slurry flow rate of 1.4 m/s. The total amount of coal gangue disposed by hanging pipe filling was 5t, the water consumption for grouting was 4.5t, each grouting time was 20 min, and the slurry flow rate was 1.5 m/s.
2. The pumping pressure and orifice pressure of roadway buried pipe filling are relatively high, and the slurry first fills the outlet space of the pipeline, which was called the slurry flowing period. As the grouting amount gradually increases, the slurry flows into the gangue gap, which was called the slurry flowing period. Due to the resistance of the gangue gap, the velocity of the slurry in the gap gradually decreases, and the slurry diffuses in the form of seepage, which was called the slurry seepage period.
3. Both burying pipes and hanging pipes for filling in the caving area can safely and efficiently fill the gangue slurry. In contrast, hanging pipes for filling cover the vertical space of the caving area, which was more conducive to the horizontal diffusion of the slurry and the accurate and efficient used of multiple types of residual space in the caving area.
4. The diffusion distance of the caving area of the working face in this test was 81 cm to 98 cm, which was basically consistent with the diffusion distance of the coal gangue slurry in the simulation test of the caving area. As the test area was a part of the entire caving area, it was equivalent to no closed boundary constraints, and the pressure less filling slurry extends in the form of a vertebral body.
5. When using buried pipe filling, there was a characteristic that the slurry accumulation thickness directly below the orifice was relatively large, and the slurry on both sides was relatively thin. At the same time, due to the small amount of filling slurry in this test, the residual space in the caving area of this test was not fully utilized, and the actual diffusion distance was lower than the theoretical diffusion distance.

## Conclusions

1. The water-to-gangue ratio was the main controlling factor for the conveying performance of coal gangue slurry. The extensibility, slump, and bleeding rate of coal gangue slurry increase with the increase of the water-to-gangue ratio, which indirectly leads to an increase in the initial and final setting time of coal gangue slurry.
2. The coal gangue slurry gradually infiltrates and diffuses to the bottom over time, indicating that the slurry had good fluidity in the gaps of scattered gangue and was suitable for grouting and filling of gangue in underground caving areas. Under the same inclination angle, the diffusion distance of the slurry increases with the increased of infiltration time. The continuous diffusion ability and diffusion speed of slurry with different inclination angles were influenced by the spatial stacking morphology of scattered gangue, and there were differences.
3. Both burying and hanging pipes in the falling area can safely and efficiently filled the gangue slurry. The diffusion distance of the caving area in the test working face was basically consistent with the diffusion distance of the slurry in the simulation test of the coal gangue slurry caving area. The research results provide data support and theoretical basis for carrying out accurate and efficient fluidized filling of gangue in caving areas in coal mines.

## Data Availability

The datasets used and/or analysed during the current study available from the corresponding author on reasonable request.
